# Tri-Party Underground Symbiosis between a Weevil, Bacteria and a Desert Plant

**DOI:** 10.1371/journal.pone.0076588

**Published:** 2013-11-11

**Authors:** Oren Shelef, Yael Helman, Ariel-Leib-Leonid Friedman, Adi Behar, Shimon Rachmilevitch

**Affiliations:** 1 French Associates Institute for Agriculture & Biotechnology of Drylands, The Jacob Blaustein Institutes for Desert Research, Ben-Gurion University of the Negev, Midreshet Ben-Gurion, Israel; 2 Department of Plant Pathology and Microbiology, The Robert H. Smith Faculty of Agriculture, Food and Environment, The Hebrew University of Jerusalem, Rehovot, Israel; 3 Department of Zoology, George S. Wise Faculty of Life Sciences, Tel-Aviv University, Tel-Aviv, Israel; RIKEN Center for Sustainable Resource Science, Japan

## Abstract

Inhabitants of arid ecosystems face severe nitrogen and water limitations. Inventive adaptations by organisms occupying such habitats are essential for survival. This study describes a tri-party symbiotic interaction between a plant (*Salsola inermis*), a beetle (*Conorhynchus pistor*), and a bacterium (*Klebsiella pneumonia*). The weevil survives by living within a mud structure affixed to the plant roots, thus benefiting from increased carbon and water, and refuge from predators and parasites. Active nitrogen-fixing bacteria harbored within the weevil's gut mediate this interaction, by supplying nitrogen to the system, which eventually promotes seed development. We studied the correlation between the weevil's existence and (i) root carbon and nitrogen content, (ii) soil water content and (iii) seed weight. Roots hosting weevils contained more nitrogen, heavier seeds and less carbon. In addition, water content was higher around the roots than in open spaces a short distance from the plant stem. Bacterial studies and nitrogen-fixation analyses, including molecular and chemical assays, indicated atmospheric nitrogen fixation in the larval stage and identified the bacterium. The coexistence of weevil and bacterial behavior coinciding with the plant's life cycle was revealed here by a long period of field observations. Out of over 60,000 known weevils, this is the only report of a weevil living most of its life underground without harming plants. The unique tri-party interaction described herein shows the important ecological role of desert plant roots and provides an example of a sustainable consortium of living organisms coping with the challenging desert environment.

## Introduction

Hot, dry environments exert challenging conditions on their inhabitants, such as the lack of water, or unpredictable water supply faced by animals and plants [1]. In addition to water scarcity, the desert environment consists of high radiation and therefore high temperatures, high salinity, strong winds, poor soil for plants [2] and poor resources for animals. Organisms use numerous strategies to escape the unfavorable conditions [3], such as seasonal dormancy [4], nocturnal or crepuscular activity [5], migration, and spatial behavior [6]. Aside from the physiological and behavioral adaptations, organisms are assisted by other organisms to survive in the desert. This study investigates how three different species improve their ability to cope with the harsh desert environment by relying on each other's advantages in obtaining water, nutrients and protection.

Nitrogen is second only to water as a limiting factor in the world's deserts [7]. Atmospheric nitrogen is inert and cannot be directly accessed by plants, which utilize mainly inorganic nitrogen, such as ammonium and nitrate. The quantity of inorganic nitrogen in desert soils is low, with seasonal variations. Nitrate, although freely available to plants, is present at only 0–4 ppm in desert soils. Indeed, the leaves of perennial desert plants contain only about 3% nitrogen, compared to about 4% in plants in more temperate regions [8], [9]. In annuals, the nitrogen content is usually even lower [8].

Through biological nitrogen fixation, some prokaryotic microorganisms can convert atmospheric nitrogen to nitrogenous compounds and in so doing, may provide their microenvironment with a more accessible nitrogen source. Symbiotic interactions between nitrogen-fixing bacteria and plants have been extensively studied and are well characterized [10]–[14]. Recent evidence of nitrogen-fixing bacteria in symbiotic interactions with arthropods ascribes an important role for these bacteria in the host's growth [15]–[18]. Recently, Behie et al. [19] showed how a soil fungus (*Metarhizium robertsii*) provides nitrogen to plants in exchange for carbon. The nitrogen source was soil microarthropods. Klironomos and Hart [20] showed that trees receive nitrogen from, and donate carbon to ectomycorrhiza that obtain nitrogen by killing small arthropods. Pinto-Tomas et al. [18] isolated symbiotic nitrogen-fixing bacteria from the fungus gardens of leaf-cutter ant colonies, and Treseder et al. [21] described how leaves of the epiphyte *Dischidia major* occupied by ants (*Philidris* sp.) contribute nearly 40% of its host plant's carbon needs through ant respiration and 30% of the plant's nitrogen demand via ant debris.

Associations between bacteria and weevils have been studied for more than a decade [22]–[26]. Studies show that most weevils carry endosymbionts within special organs [27]. Login et al. [28] recently elucidated the mechanism regulating the growth of the primary endosymbiotic bacteria (SPE) residing within bacteriomes in the gut of *Sitophilus* spp. weevils. Toju and Fukatsu [27] recently published a comprehensive survey of endosymbionts in natural populations of the chestnut weevil *Curculio sikkimensis*. They identified six distinct endosymbiont lineages, namely *Curculioniphilus*, *Sodalis*, *Serratia*, *Wolbachia*, *Rickettsia* and *Spiroplasma*, at different infection frequencies and with different geographical-distribution patterns.

In many desert ecosystems, plant communities are dominated by annual species, which typically exhibit rapid wet-season growth followed by dormancy or decay during the dry summer months [29], [30]. An exception to this general rule is *Salsola inermis*, an annual shrub that flourishes during the dry summer in saline soils [31], and the only visibly green annual in the Negev Desert of Israel. The weevil *Conorhynchus pistor* (see Figure S1 and Movies S1, S2 for photos and a detailed description) lives on this plant's roots in attached mud chambers. Here we investigated the unique underground relationship between this plant and weevil, and the bacteria that live inside the weevil's gut. The main hypothesis was that the plant provides the beetle with nutrition and amenable climatic conditions, and the weevil contributes nitrogen to the plant via nitrogen-fixing symbionts.

The overall goal of the current research was to study and describe the novel finding of this tri-party desert symbiosis that might be encountered in many other ecosystems and might contain a key to sustainability in hot deserts. Uncovering the hidden relationship between organisms, as in this system, reveals the complexity of environmental adaptations within the community, rather than in only one distinct species *per se*.

## Materials and Methods

### Research Site

*S. inermis* is distributed all over the Negev Desert. The study was conducted in the Sede Zin plateau in the Central Negev Highlands, Israel (30°51′N, 34°47′E). The Negev region is characterized by hot, dry summers with an average maximum daily temperature of 32.7°C in July–August and a cold, wet winter with 99 mm annual precipitation, and average minimum daily temperature reaching 4.6°C in January–February (data from the Israel Meteorological Service). Host plants were found in loose sandy loam soil.

### Life-History Studies

The weevil's life cycle was observed mainly in the field. Attempts to mimic the conditions in a controlled terrarium met with limited success, providing a poor statistical data set. Life-cycle phases were estimated based on 5 years of root excavations and field observations. No specific permissions were required for these locations or activities since they were performed in open spaces around Ben Gurion University and field studies did not involve endangered or protected species. To find *S. inermis* specimens hosting weevils with mud structures, we gently excavated a natural population of the plants with a shovel and then compared host plants to neighboring nonhost plants. Our estimation, based on counting host and nonhost plants in the last excavation session, is that about 5% of the *S. inermis* population harbored hosting beetles. Excavations were performed from June 2007 to January 2012. Observations were made in terrariums placed in a controlled-temperature greenhouse set at 25°C. Adult weevils were excavated in the field and transferred to the terrarium with young seedlings of *S. inermis*, which had been seeded and sprouted in the terrarium in advance. The main challenge was to keep the soil humid enough but not too muddy. Weevils were sexed by genitalia analysis and morphology, and care was taken to have males and females in the same terrarium. Reports are limited because all plants and beetles died before oviposition occurred.

### Carbon and Nitrogen Analyses

To trace the fate of the nitrogen and carbon compounds in the weevil–plant system, total nitrogen levels were compared in *S. inermis* plants with and without weevils attached to their roots. For elemental analyses, host plants were compared to the nearest nonhost plants, at a distance of 5–50 cm. Plant samples were dried (65°C for 48 h) and pulverized, and 2.7 mg of each sample was analyzed by a FlashEA™1112 CHNS-O Analyser (Thermo Fisher Scientific Inc., UK); 16 pairs of host and nonhost *S. inermis* plants were analyzed.

### Seed Weight

To quantify the effect of the weevils' presence on seeds in terms of fecundity, seed weight of *S. inermis* plants with and without weevils attached to their roots was measured (CPA Analytical Balance CPA225D, Sartorius AG, Germany). Eight plant pairs were sampled, for a total of 156 seeds.

### Soil Water Content

To study the plants' impact on microclimatic conditions, soil water content was measured around the *S. inermis* plants. Soil for water content analysis was sampled at fixed distances of 0, 5 and 20 cm from the plant's stem (at 0–10 cm depth). Measurements were performed with nine plants. Soil water content was measured gravimetrically before and after oven-drying (105°C for 48 h).

### Bacterial Studies and Nitrogen Fixation

Due to the extremely low levels of nitrogen in such arid ecosystems, we hypothesized that the source of the higher nitrogen content in the hosting plants is atmospheric nitrogen gas reduced to ammonia as a result of nitrogen fixation by symbiotic bacteria. A widely used functional test – acetylene reduction assay – was performed to determine whether the nitrogen enrichment detected in the roots of the host plants could be due to nitrogen fixation by symbiotic bacteria. The acetylene reduction assay detects the active nitrogenase enzyme complex used by nitrogen-fixing prokaryotes: the nitrogenase enzyme is capable of reducing the triple bond in acetylene, yielding ethylene, which is then detected by gas chromatography [11]. Acetylene was injected into closed vessels (15 ml) containing one weevil (adult or larva) to a final concentration of 20% (v/v) by replacement of an identical volume of air. A total of 16 vessels containing weevil larvae and 16 vessels containing weevil adults were analyzed. Autoclaved weevils were used as a negative control (five adults and five larvae). Reduction of acetylene to ethylene was measured by flame-ionization gas chromatography (Thermo Fisher Scientific Inc. FOCUS GC) after 2, 4 and 6 h of incubation at 25°C.

To identify the potential nitrogen fixers, DNA samples were obtained from both adult and larval weevil. DNA samples from three adult weevils and three larval weevils (In total, there were three replicates from each developmental stage) were subjected to mass sequencing. 16S rDNA bacterial tag-encoded FLX amplicon pyrosequencing was performed by the Research and Testing Laboratory (Lubbock, TX, USA) as described by Dowd et al. [32]

Amplicons originated from the 27F region (numbered relative to *Escherichia coli* rRNA). In addition, DNA extracted from five adult weevils and five larval weevils was searched for the *nifH* gene encoding the small subunit of the evolutionarily conserved nitrogenase enzyme complex. PCR amplification was performed using a primer set targeted to Enterobacteriaceae species (expected product size 660 bp): NH1f (5′-ACACCATTATGGAGATGG-3′); NH1r (5′-GATGCCGAACTCCATCAG-3′) according to Behar et al. [17]. PCR Products were then sequenced using an ABI 3730xl DNA Analyzer for DNA sequencing by the Hylab Co. sequencing service. Phylogenetic analysis of the *nifH* gene was then implemented in PhyML [33].To assess confidence in nodes, 100 bootstrap replicates were performed. Sequences obtained from *Azotobacter vinelandii* strain DJ were used as the outgroup. Accession Numbers (NCBI) - The nucleotide sequences of the *nifH* gene were deposited in the GenBank database under accession numbers JX406864–JX406865.

### Statistics

Data sets that fit the requirements for statistical analysis were tested for Gaussian distribution by Shapiro–Wilk test. For those in which a Gaussian distribution was detected, the parametric Student's t-test or Tukey HSD was used to check for significant differences. Nonparametric Mann–Whitney U-test was used whenever the data set did not fit the requirements of normal distribution. Calculations were conducted with SAS 10 (SAS Institute, Cary, NC, USA). Number of repetitions (n) and the statistical procedure performed for each measurement are reported.

## Results

### Life-History Studies

**Figure 1:**
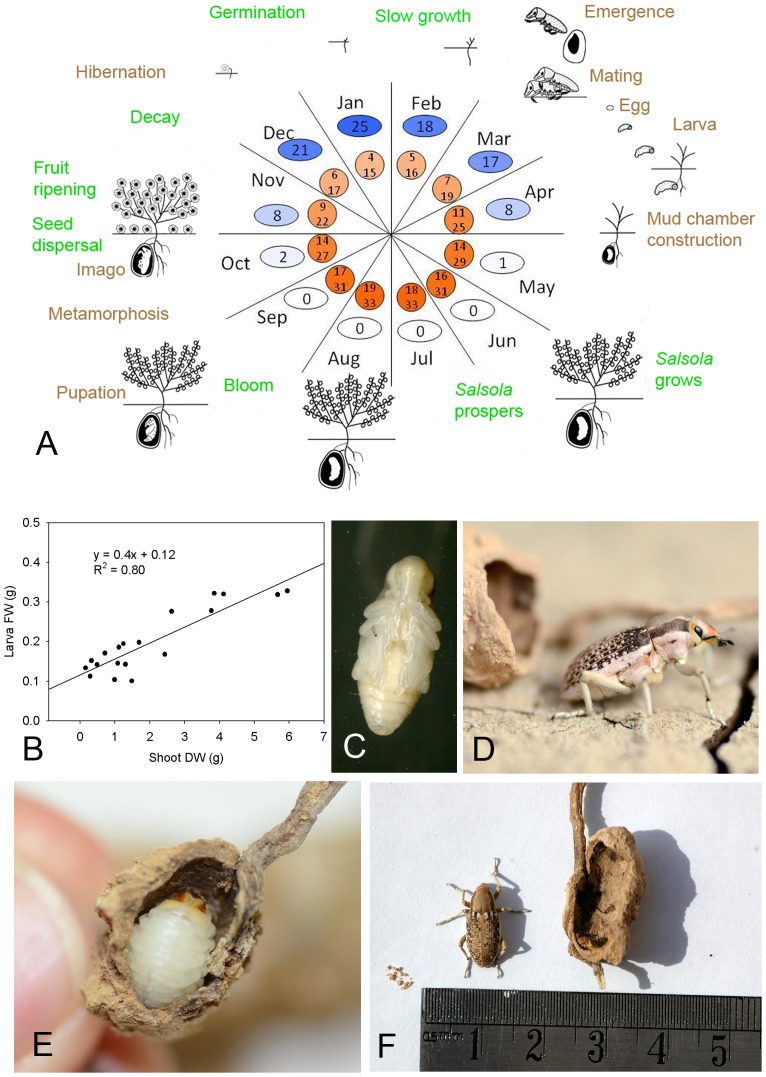
Combined plant–beetle life cycles. (A) Illustration of the co-occurring life cycles. Green = life-cycle phase of the desert plant *Salsola inermis*; brown = life-cycle phase of weevils (*Conorhynchus pistor*) that build mud chambers on *S. inermis* roots; ellipse = monthly average accumulated precipitation; circle = minimum and maximum daily temperatures. (B) Correlation between weevil larva fresh weight (FW) and its host plant's dry weight (DW) (n = 19). (C) *C. pistor* pupa. (D) *C. pistor* imago and mud chamber. (E) *C. pistor* larva in mud chamber. (F) *C. pistor* imago near its mud chamber on *S. inermis* root. Pupa slough is seen at the lower part of the mud chamber.

Based on observations of weevils in their natural environment from 2007–2012 and to some extent in controlled-environment terrariums, a proposed scheme is given for their life cycle (Figure 1, Movies S1 and S2). Observations indicate that weevil development is correlated with plant growth (Figure 1B). The larva builds its mud chamber attached to the roots while the plant is thriving and when the plant starts to decay, the larva pupates. The weevil in its subsequent imago stage hibernates underground in the cold winter and hatches in the spring when temperatures rise. Adult weevils can stay in their mud chambers for extended periods before emerging. Indeed, adults in the laboratory survived for more than 5 months in a mud chamber, and in the field, live adults were found on dead plants from the previous year, indicating that the beetles aestivated in the summer and remained viable for over a year in their mud chambers.

### Effect of Weevils on Carbon and Nitrogen Content, Seed Weight and Soil Water Content

**Figure 2:**
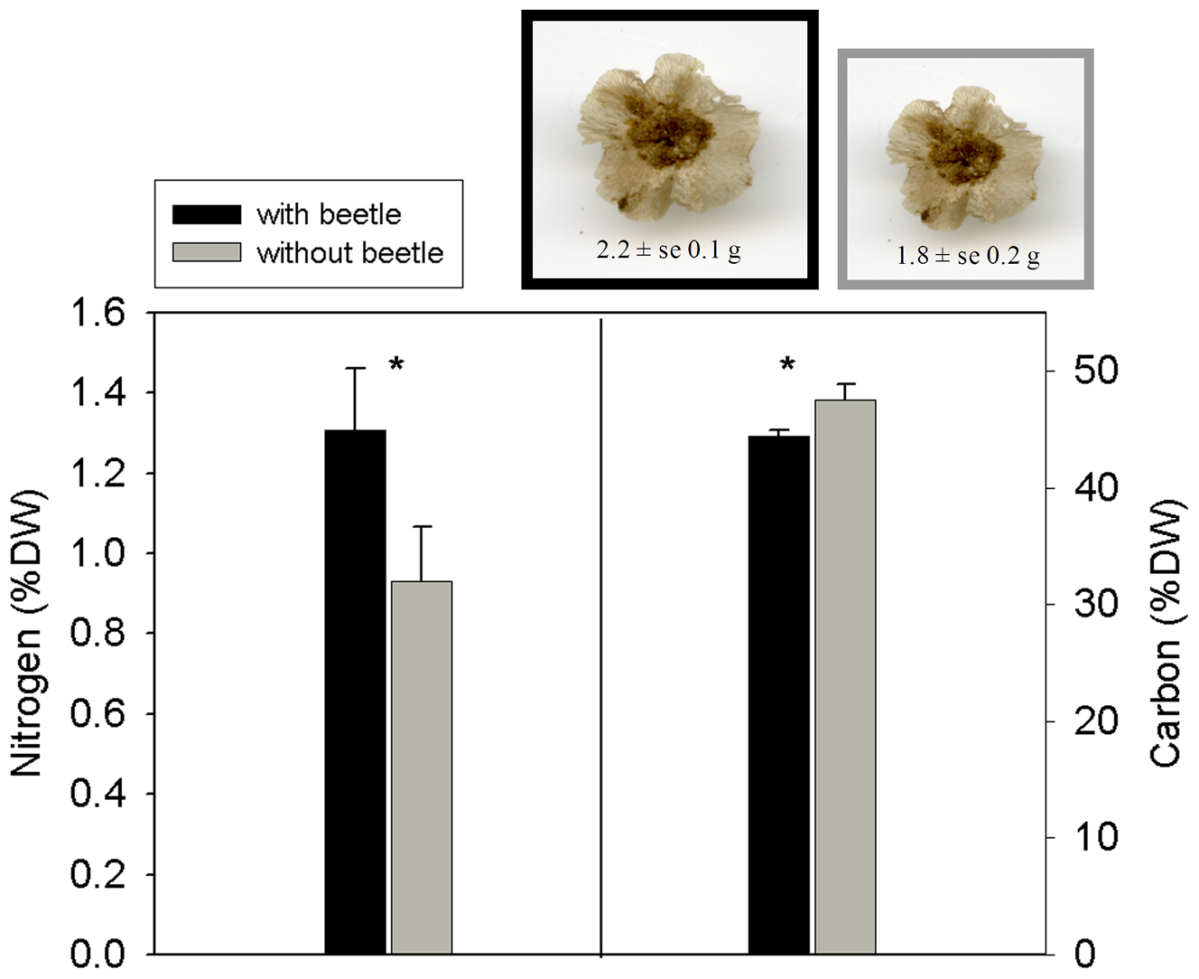
Nitrogen and carbon contents in host *Salsola inermis* vs. nonhost plants and effect on seed size. Roots were measured at the mud-chamber-attachment points; seed weight ± SE is given on the seed images; photos were taken at the same magnification for host and nonhost seeds– size difference is only for illustration. %DW is percentage of dry weight. Error bars denote SE. Significant differences are indicated by an asterisk. For % nitrogen, Student's t-test value = 2.39, df = 30, *P* = 0.023, n = 16; for % carbon, Mann–Whitney U-test value = 61, *P* = 0.034, n = 15.

**Figure 3:**
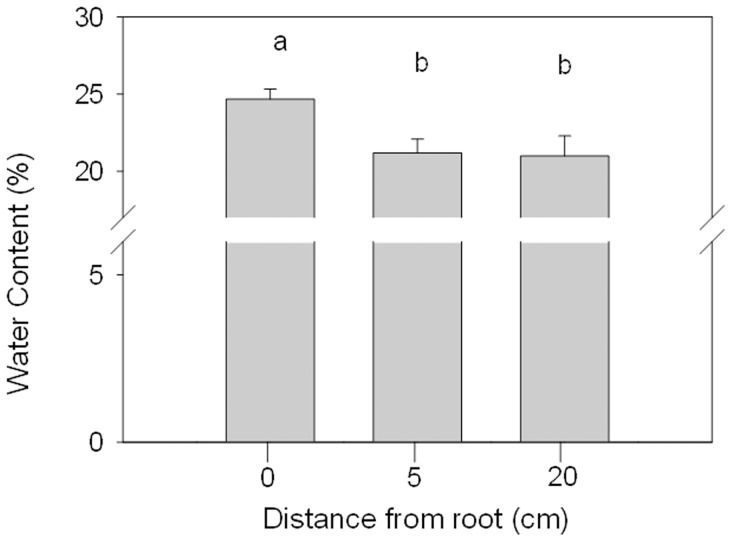
Effect of proximity to roots on soil water content. Error bars denote SE. Different letters denote significant differences (Tukey HSD test, MS = 6.26, df = 32, *P*<0.05, n = 9).

The roots of host plants contained 29% more nitrogen (mean ± SE 1.3±0.15%) and 7% less carbon (44.4±0.51%) than those of nonhost plants (0.93±0.14% nitrogen, 47.56±1.3% carbon, n = 16 plant pairs) (Figure 2). The seeds of the host plants were 17±0.19% heavier than those of nonhost plants (Student's t- test, *P*<0.001, n = 8) (Figure 2). Moreover, the soil water content beneath *S. inermis* plants was 15% higher 24.7±1.83% vs. 21.1±3% than in nearby open spaces (Figure 3).

### Bacterial Studies and Nitrogen Fixation

**Figure 4:**
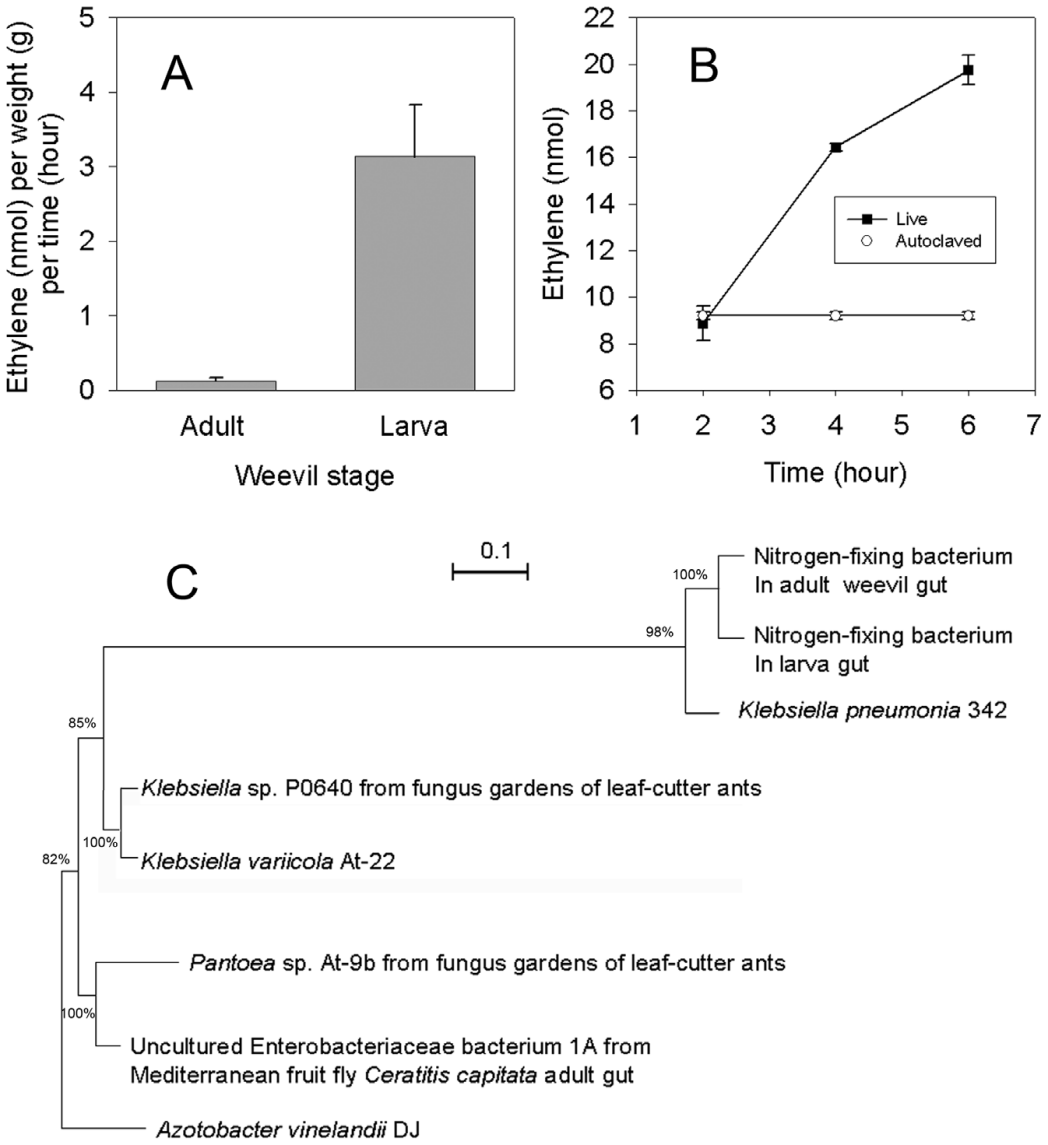
Evidence of symbiotic nitrogen-reducing associations between weevils and bacteria. (A) Nitrogen-fixing activity measured by acetylene reduction assay for adult weevils and larvae; rates calculated after blank subtraction of autoclaved samples (one-way ANOVA, F = 8.61; *P*<0.001, n = 16). (B) Amount of ethylene accumulated over time in live weevil larvae compared to autoclaved larvae as a control. (C) Rooted maximum-likelihood phylogenetic tree based on *nifH* sequence analysis of nitrogen-fixing bacteria associated with the weevil *Conorhynchus pistor*. Scale bar indicates 10% estimated sequence divergence. *Azotobacter vinelandiii* was used as the outgroup. Values above and below branches indicate likelihood bootstrap support. Only bootstrap values greater than 70% are shown.

Acetylene-reducing activity was detected in live weevil larvae but not in adult weevils (3.135±0.7 and 0.12±0.05 nmol ethylene h^−1^ weevil weight^−1^, respectively; one-way ANOVA: F = 8.61; *P*<0.001; n = 16) (Figure 4A). Measurements over a period of 6 h showed that ethylene accumulated over time in live weevil larvae but not in autoclaved dead larvae, providing direct evidence of nitrogen fixation occurring *in vivo* in weevil larvae (Figure 4B).

Sequence analysis of *nifH* suggested that the nitrogen-fixing bacterium residing in the weevil is *Klebsiella pneumonia* (Figure 4C). Although nitrogen fixation was not detected in adult weevils, the *nifH* gene was detected in both adult and larval DNA samples. Furthermore, bacterial community structure and composition of both adult and larval weevils were determined by using mass sequencing of 16S rRNA gene fragments. A total of 98 and 85 bacterial sequences were obtained and analyzed from larvae and adults respectively. In accordance with *nifH* analysis, *Klebsiella* spp , were the only known nitrogen fixing bacteria found within the insects (∼5% from the total bacteria community in both developmental stages). Other lineages found were originated mainly from: *Pseudomonas* spp. (40%); *Escherichia* spp. and *Staphylococcus* spp. (∼5% each); *Wolbachia*, *Acinetobacter* and *Providencia* (∼1% each) in larvae; and *Escherichia* spp. (75%) and *Trabulsiella* spp.(17%) in adults.

## Discussion

Findings provide evidence for the mutuality of the unusual relationship between a desert plant, a weevil, and nitrogen-fixing bacteria. Of the more than 60,000 known weevil species, *C. pistor* is the only documented weevil that spends most of its life cycle underground. Our 5% colonizing rate estimation of weevils per shrubs might be an underestimation, since some beetles are likely to remain in the ground even when the plant is uprooted. Findings from both *nifH* and mass sequencing analyses showed that *C. pistor* harbors nitrogen-fixing bacteria of the genus *Klebsiella*. Even thought, these symbionts were found in both larvae and adults, they were shown to fix nitrogen only during the underground larval phase (Figure 4A), which is nourished by carbon in the root exudates, and not during the imago stage, when the weevils are not dependent on exudates and can undergo long periods of fasting. Accordingly, reduced nitrogen is transferred to the plant during the weevil's larval stage at the attachment point of its mud chamber (Figures 2, 4), while the roots remain undamaged and completely functional. Results indicate that *S. inermis* contains relatively low nitrogen levels, further supporting the paradigm that nitrogen is a major limiting factor under desert conditions [7]. Nonetheless, weevil-hosting plants had increased nitrogen and decreased carbon levels, resulting in a lower carbon-to-nitrogen ratio than in nonhost plants. The relatively low level of carbon detected in the host plants (Figure 2) is explained by its exploitation by the larvae. This was further corroborated by observations of premature pupation in young excavated plants. In this case, presumably, the arrest of the carbon supply to the larvae, due to cutoff of the carbon-assimilation source, led to the emergence of dwarfed adults (S1E). The lower carbon-to-nitrogen ratio promoted enlarged seeds, as reflected by their higher weight (Figure 2). Thus, the presence of *C. pistor* and its nitrogen-fixing bacteria raised *S. inermis* nitrogen levels and positively affected plant reproduction. A positive effect of free-living, nitrogen-fixing *Azospirillum* spp. bacteria on the reproductive yield of plants has been previously demonstrated by Okon et al. [34].

Results support the hypothesis that weevils enjoy an improved microclimatic environment, with higher water content, in proximity to the root (Figure 3). These results correspond with those of a recent study showing that water content in the root zone of plants is higher than that in the surrounding soil [35]. In return, the beetle hosts free-living nitrogen-fixing bacteria in its gut, which supply nitrogen to the roots. To the best of our knowledge, the current study is the first to report such a mutualistic tri-party interaction in a desert environment, specifically as practiced by *C. pistor* which grows and hibernates underground and occasionally aestivates in mud chambers on the plant roots.

Associations between *Salsola* spp. and weevils have been previously reported; however, those studies described the harmful effects of the beetles on the plants [36], [37]. For example, *C. faldermanni* adults have been found in Middle Asia on *Salsola* spp. roots [38]. The larvae of *Menecleonus lagopus*, from the same tribe as Cleonini, pupate while attached to the roots of *Salsola carinata* where the imago overwinters. Imaginal stages of other related genera also overwinter underground [38].

To date, there are only three other known examples of biological nitrogen fixation within Insecta- termites, the Mediterranean fruit fly and leaf cutter ants. All these symbioses were shown to play an important role in micro-environment in which they occur [17], [18], [39]. Our results provide evidence of the important role that this tri-party symbiosis has within the desert ecosystem. In this hot, dry environment, living underground entails a definite advantage. The symbiotic tri-party interaction described herein indicates the important ecological role of desert plant roots, as well as that of underground insects as a reservoir for plant-growth-promoting bacteria. Exploration of the weevil's life history on the roots of *S. inermis* exemplifies the importance of underground life in the desert and, specifically, the ecological role of roots, even during plant decay.

## Supporting Information

Figure S1 **Photos of** ***Conorhynchus pistor*** **(A–E) and** ***Salsola inermis*** **(F).** *C. pistor* Chevrolat is a ground-dwelling weevil that is highly variable in body size, ranging from 0.8–1.6 cm, usually between 1.0 and 1.3 cm. It has a short rostrum that tapers at the apex, and its body and appendages are covered by a thick layer of whitish, creamy, yellowish, or brown oblong scales. These are arranged in a pattern of distinct white and brown longitudinal bands on the lateral parts of the pronotum and elytra, while the median part of the elytra is a whitish-grayish brown, either without a pattern or tessellated. Distribution: Syria, Iran [28] and Israel (Judean Desert, Dead Sea area, Negev, Arava Valley). Photos of *C.pistor* taken by Oz Rittner. Photo F in figure S1 and photos C–F in Figure 1 were taken by Oren Shelef. Figure 1A illustrations by Ariel-Leib-Leonid Friedman.
(TIF)

Movie S1 **Larva building mud chamber (40 s).**
(MP4)

Movie S2 **Beetle hatching (46 s).**
(MP4)
