# Supplementary figures and images for: Tri-Party Underground Symbiosis between a Weevil, Bacteria and a Desert Plant
Source: PLoS One. 2013 Nov 11;8(11):e76588. doi: 10.1371/journal.pone.0076588 (PMC3823913; doi:10.1371/journal.pone.0076588)

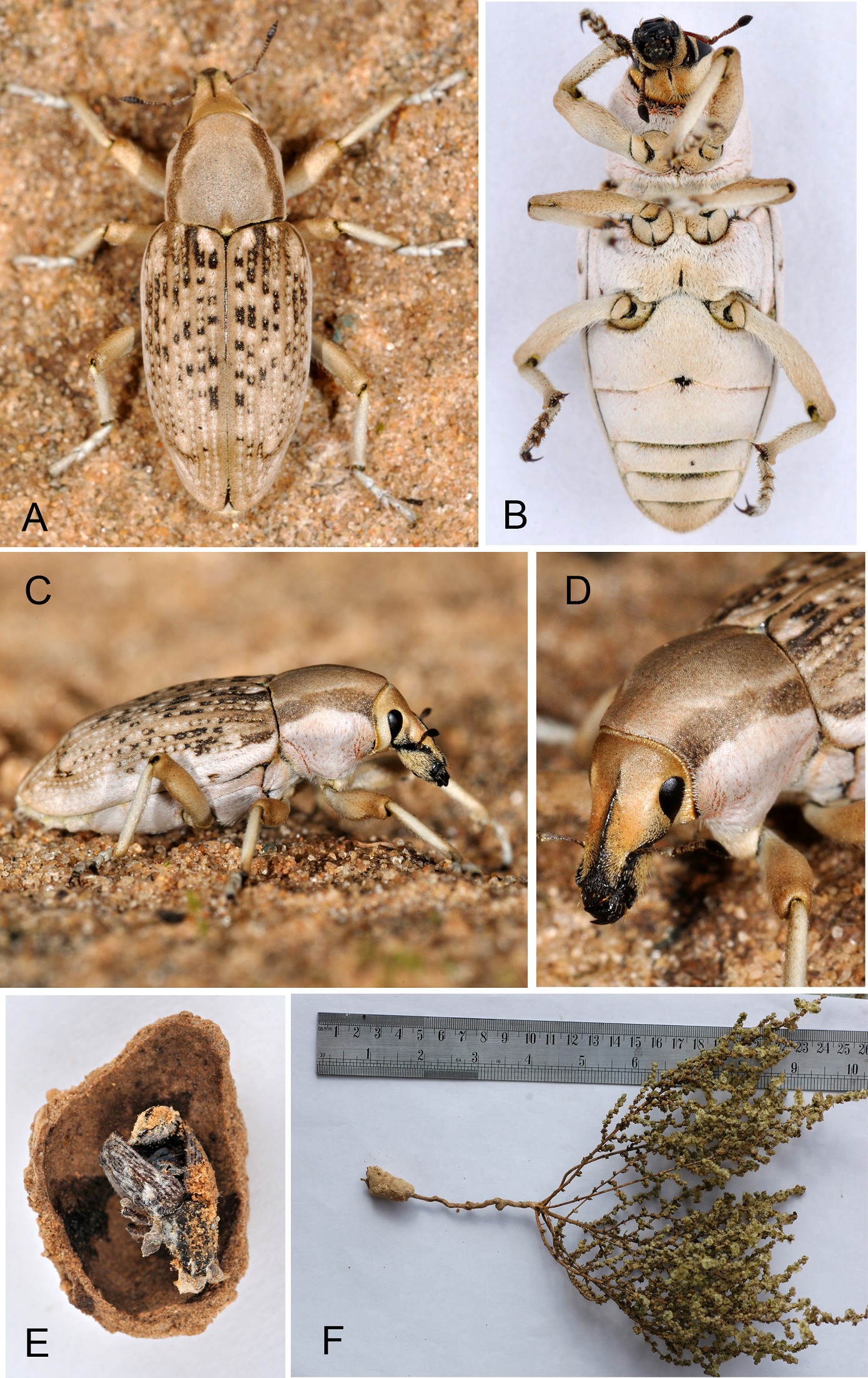

Supplement: Figure S1 — Photos of Conorhynchus pistor (A–E) and Salsola inermis (F). C. pistor Chevrolat is a ground-dwelling weevil that is highly variable in body size, ranging from 0.8–1.6 cm, usually between 1.0 and 1.3 cm. It has a short rostrum that tapers at the apex, and its body and appendages are covered by a thick layer of whitish, creamy, yellowish, or brown oblong scales. These are arranged in a pattern of distinct white and brown longitudinal bands on the lateral parts of the pronotum and elytra, while the median part of the elytra is a whitish-grayish brown, either without a pattern or tessellated. Distribution: Syria, Iran [28] and Israel (Judean Desert, Dead Sea area, Negev, Arava Valley). Photos of C.pistor taken by Oz Rittner. Photo F in figure S1 and photos C–F in Figure 1 were taken by Oren Shelef. Figure 1A illustrations by Ariel-Leib-Leonid Friedman. (TIF) [file pone.0076588.s001.tif]
